# Evaluating Human-Computer Co-creative Processes in Music: A Case Study on the CHAMELEON Melodic Harmonizer

**DOI:** 10.3389/fpsyg.2021.603752

**Published:** 2021-02-22

**Authors:** Asterios Zacharakis, Maximos Kaliakatsos-Papakostas, Stamatia Kalaitzidou, Emilios Cambouropoulos

**Affiliations:** School of Music Studies, Aristotle University of Thessaloniki, Thessaloniki, Greece

**Keywords:** creativity support tools, creativity evaluation, melodic harmonization, musical harmony, conceptual blending

## Abstract

CHAMELEON is a computational melodic harmonization assistant. It can harmonize a given melody according to a number of independent harmonic idioms or blends between idioms based on principles of conceptual blending theory. Thus, the system is capable of offering a wealth of possible solutions and viewpoints for melodic harmonization. This study investigates how human creativity may be influenced by the use of CHAMELEON in a melodic harmonization task. Professional and novice music composers participated in an experiment where they were asked to harmonize two similar melodies under two different conditions: one with and one without computational support. A control group harmonized both melodies without computational assistance. The influence of the system was examined both behaviorally, by comparing metrics of user-experience, and in terms of the properties of the artifacts (i.e., pitch class distribution and number of chord types characterizing each harmonization) that were created between the two experimental conditions. Results suggest that appreciation of the system was expertise-dependent (i.e., novices appreciated the computational support more than professionals). At the same time, users seemed to adopt more explorative strategies as a result of interaction with CHAMELEON based on the fact that the harmonizations created this way were more complex, diverse, and unexpected in comparison to the ones of the control group.

## 1. Introduction

The desire to define and measure human creativity (e.g., Stein, 1953; Rhodes, 1961; Mooney, 1963; Boden et al., 2004; Wiggins et al., 2015) or even further to identify neural underpinnings of creative behaviors (e.g., Rosen et al., 2020; Boccia et al., 2015; Luft et al., 2018) has a long history in which music has had a prominent position (e.g., Johnson-Laird, 1988; Odena and Welch, 2009; Boccia et al., 2015; Rosen et al., 2020). Recent advances in artificial intelligence have made computational creativity a rapidly emerging scientific field. The general objective of this interdisciplinary research area is to obtain a deeper understanding and modeling of human creative processes in order to produce creative systems that can either exhibit creativity of their own, or assist humans in becoming more creative (e.g., Wiggins, 2006, 2008; Colton and Wiggins, 2012; Jordanous, 2012; Agres et al., 2016). This has, in turn, mandated the rigorous evaluation of artificial creativity which, like the evaluation of human creativity, poses a challenging problem.

Creative systems are usually evaluated either with respect to their end products (e.g., Ritchie, 2007) or the processes they employ to reach them (Colton, 2008). More recently some extra considerations were revisited as part of the older four Ps framework for creativity evaluation (Jordanous, 2016). According to the four Ps, the creative producer (i.e., the computer software or indeed the human programmer) and the press (i.e., the environment in which a creative act takes place) should also be added to the product and process criteria for a more comprehensive assessment of computational creativity. The evaluation of computational creativity in the arts becomes even more complex mainly due to the lack of a clear-cut metric for measuring success or failure. Unlike other fields of artificial intelligence (e.g., game-playing, computer vision, etc.), the generation of an aesthetic artifact does not have a strictly defined goal (such as winning a game of chess), thus making the assessment of its merit a rather challenging problem. Therefore, breaking down creativity into several—easier to assess—constituent dimensions such as novelty, divergence, value, problem solving ability, etc., constitutes a reasonable approach for the evaluation of creative systems (e.g., Jordanous, 2012).

At the same time, computational systems can be exploited as tools for enhancing human creativity in addition to being autonomous creative agents. In such a case, a computational system should be assessed in terms of the potential it offers for creativity support rather than its absolute creativity *per se*, i.e., the spotlight should be redirected from measuring how creative the actual system is, to measuring how it may enhance the creativity of a human user. The interest in the impact of technology on human creativity is more recent (Lubart, 2005; Shneiderman et al., 2006; Shneiderman, 2007) in comparison to research on the definition and evaluation of creativity itself (see, Cherry and Latulipe, 2014). However, creativity support has already been studied in the context of various creative human activities such as poetry writing (Kantosalo and Riihiaho, 2019), creative design (Albert and Runco, 1999; Bonnardel and Zenasni, 2010), 3D modeling (Chaudhuri and Koltun, 2010), or general problem solving (Massetti, 1996).

Artificially generated music is often limited to the level of style imitation, a task in which artificial intelligence methods become increasingly competent; this is achieved either by employing “traditional” rule-based methods (Ebcioğlu, 1988), Hidden Markov models (Allan and Williams, 2005; Raczyński et al., 2013) or, more recently, machine learning techniques based on artificial neural networks (see Briot et al., 2017). Successful style replication is considered in certain respects a creative task and advanced techniques have exhibited interesting results toward this direction (e.g., Hadjeres et al., 2017). However, departing from a given style into new unexplored musical territory has often a greater creative value. Attempts have been made to “interpolate” between, or even “extrapolate” from the learned material and generate music that either meaningfully crosses the borders of learned styles (e.g., Roberts et al., 2017), or applies stylistic aspects of one learned style to another (Brunner et al., 2018). The methods and approaches reviewed herein, are the most relevant to what CHAMELEON was designed and developed for. For a thorough review of the many methods and approaches that have been successfully applied to style-related music generation, the reader is referred to Kaliakatsos-Papakostas et al. (2020).

The focus of this paper is on the CHAMELEON^1^ melodic harmonization assistant (Kaliakatsos-Papakostas et al., 2017), which follows a paradigm of computational creativity that not only extrapolates musical styles, but also generates fundamentally new harmonic material through hybrid methods that are based on generative implementations of Conceptual Blending (CB) and statistical learning. CB Theory has been examined as a fundamental tool that humans use to understand and generate new concepts (Fauconnier and Turner, 2003; Goguen, 2006), whereby two *input* conceptual spaces are combined to generate a new conceptual space. The new conceptual space commonly features new unforeseen properties that arise from the combination of elements and relations of the input spaces. CHAMELEON employs a generative algorithmic implementation of CB theory on chord transitions, where chords are represented by their General Chord Type (GCT, Cambouropoulos et al., 2014). The GCT incorporates an algorithm that first performs root finding on a chord and then it unrolls the hierarchy of the remaining pitch classes, identifying the basic components of this chord (i.e., third, fifth, seventh, and other extensions); we will be referring to these basic components as the “type” of the chord. Chord transitions in CHAMELEON (pairs of successive chords), are modeled as pairs of GCTs, along with information about the root motion of the involved chords (integer value between −5 and 6) and whether there is semitone motion (ascending or descending) to the root of the second chord (separate boolean values for ascending and descending). Training data of GCT transitions from diverse styles are fed into CHAMELEON which learns transition probabilities and can generate new melodic harmonizations from the learned styles using a Hidden Markov Model. Therefore, the transition probability matrix describes chord transitions, where chords are represented as GCTs.

The unique feature of CHAMELEON, however, is that it can augment the Markov transition tables of two learned idioms by blending the most common transitions of the two input idioms. The new, augmented transition probability table incorporates diagonally-adjacent copies of the initial transition matrices learned from the two idioms to be blended; it should be noted that the two learned idioms may include identical chords (e.g., the I or V^7^ chords can be found both in the Bach Chorales and in Jazz), but in the augmented transitions matrix they are considered as separate chords. The, initially empty, two anti-diagonal blocks of the augmented transitions matrix are firstly filled with probability values belonging to transitions that are identical in both blended idioms (e.g., perfect cadences can be found both in Bach Chorales and in Jazz). The probability value for each activated transition in the anti-diagonal blocks is the average of the two probabilities in the initial matrices. At a second stage, all pairs of the most common transitions on the initial idioms are blended (Eppe et al., 2015), giving rise to new chord transitions that might potentially incorporate new chords, in a sense that these chords do not belong to any of the learned idioms. Such chords are appended in the augmented matrix (a new line and a new column are added for any new chord), while the probability assigned to the transitions generated by blending is the average probability of the input transitions (for more information please refer to Kaliakatsos-Papakostas et al., 2017).

As a result, CHAMELEON can harmonize a given melody according to a number of different harmonic idioms or/and their harmonic blends^2^. This makes it capable of offering a variety of novel and unexpected “solutions” for melodic harmonization that could potentially influence human composers toward creating their own version.

From the above, it is evident that CHAMELEON can be regarded as both an autonomous computational creativity system and a creativity support system. While our previous work has investigated the former attribute of CHAMELEON by evaluating its creativity through its products (Zacharakis et al., 2018), the current work assesses CHAMELEON's performance as a creativity support tool in the domain of music. This requires a method capable of capturing a potential influence of the system on music creation by human users.

To this end, we devised an experiment to assess the way human users actively utilized the melodic harmonization assistant. Following a type of repeated-measures experimental design, one group of music students and one group of professional composers of contemporary music were initially asked simply to harmonize a given melody without any sort of influence. Subsequently, the same task was repeated on a very similar—but not the same—melody while giving participants the opportunity to interact with CHAMELEON. The aim of this evaluation was twofold: firstly to quantify user experience—also with respect to expertise—through a number of post-task questions assessing aspects related to creative behavior; and, secondly, to compare the outcome harmonizations between the two experimental conditions through computational extraction of harmonic features. The repeated-measures design was complemented by a between-groups comparison in which a different control group of novice participants performed the same two tasks but without computational support. This way, we were able to test for possible order effects in the characteristics of the produced harmonizations of the main experiment.

In order to be able to evaluate creativity in a meaningful way, a definition of this multifaceted concept is required. As discussed previously this is not a trivial problem. However, a common ground of many existing definitions is that creativity refers to a process that generates ideas or artifacts both novel (i.e., original or unexpected) and valuable (i.e., useful or appropriate) (for an overview please refer to Jordanous and Keller, 2016). This simple working definition of creativity is also adopted for the purposes of this study. Our basic hypothesis regarding the use of CHAMELEON is that it might stimulate the users toward producing more unconventional (i.e., novel) solutions compared to their initial harmonizations on very similar melodic material. It could then be argued that by creating something novel for themselves they will have manifested personal or psychological creativity (P-creativity) as defined by Boden et al. (2004). Besides, departure from the habitual thinking patterns that promotes originality has been widely deemed an important aspect of creative behavior (e.g., McCrae, 1987; Runco and Acar, 2012; Luft et al., 2018).

The assessment of whether such explorative behavior can indeed be recognized through the outcome harmonizations requires the ability to compare between different harmonic sequences of the same or very similar melodic material. While there exist a limited number of studies proposing metrics for chord distances and harmonic similarity (e.g., De Haas et al., 2008, 2010, 2011) the comparison between chord progressions is far from being a solved problem. This is particularly true for the case where harmonic progressions do not belong to standard common-practice tonal harmony (Lerdahl, 2004; Kostka and Payne, 2013). The comparison of chord sequences that belong to non-standard tonal styles or even belong to different non-tonal styles is a challenging task due to the difficulty of forming idiom-independent theories of harmony. To circumvent this problem three harmonic features (number of GCTs, number GCT types, and Pitch Class Profiles) that can either be used as descriptors of harmonic content or be transformed into general distance metrics between chord sequences in a style-independent manner were employed (see section 2.3). A preliminary analysis based on these metrics that was recently presented (Zacharakis et al., 2020) provided some evidence to support the basic hypothesis of increased harmonic diversity as a result of interaction with CHAMELEON.

The next section presents the details of the experiment, the behavioral creativity metrics used and the calculation of the harmonic features. The results section is separated into the analysis of the behavioral data and the analysis of the actual harmonizations generated by the participants. The discussion offers some perspective on the current findings and concludes by a brief reference to a compositional project that came as a byproduct of this laboratory experiment. In this project, a small subgroup of our participants were asked to compose short pieces for a string quartet that were inspired by their interaction with the system during the experiment. The pieces were presented in a live concert where each of the composers explained how they integrated ideas suggested by CHAMELEON in their compositional practice.

## 2. Materials and Methods

### 2.1. Main Experiment

**Figure 1:**
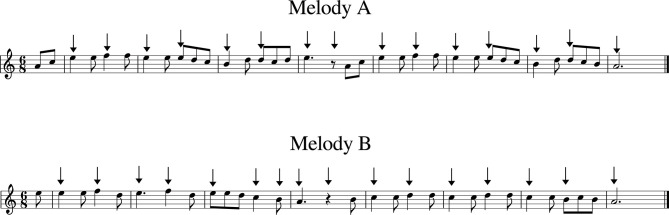
The two melodies used in the harmonization task. The upper melody (“Menexedes kai Zoumboulia”) was employed in the simple harmonization task whereas the lower one (“Lullaby from Southern Italy”) was used for the computationally-supported harmonization. Arrows indicate the requested harmonic rhythm.

Twenty five participants that were either students from the School of Music Studies of the Aristotle University of Thessaloniki (*N* = 20, mean age = 23.2, std age = 5.8, 10 female) or professional contemporary music composers (*N* = 5, mean age = 32.6, std age = 11.1, 1 female) took part in the main experiment. The experimental procedure comprised two phases in a repeated-measures design. In phase one, participants were asked to harmonize the melody of a Greek traditional folk song called “Menexedes kai Zoumboulia” (melody A) in minor mode (see Figure 1). They were asked to place chords at the positions indicated by arrows (i.e., harmonic rhythm was fixed) and to use satisfaction of personal preference as the sole criterion for their harmonization, even at the cost of not conforming to standard harmonic rules. Voice leading was not at the center of this study, therefore participants were advised to omit it in order to save time.

In the second phase, participants were similarly asked to harmonize a melody of a folk lullaby from Southern Italy (melody B) also in minor mode (see Figure 1). An inspection of Figure 1 reveals that the selected melodies of both phases were almost identical in rhythmic and harmonic features. The melodic lines were akin, the harmonic rhythms requested were almost the same and both featured very similar implied harmony (see Supplementary Figure 1 for a presentation of the most typical harmonizations for these two melodies). The selection of these two closely related melodies served the purpose of requesting an equivalent but not identical task between the two experimental conditions. The directions regarding harmonic rhythm and voice leading were identical with phase one. This time, however, participants had additional access to CHAMELEON and they were prompted to explore its capability to offer various harmonizations on this particular melody. After giving a short demonstration of CHAMELEON, the experimenter made it clear that the extent to which participants should exploit the solutions offered by it for their own harmonizations would be totally up to them. It was particularly stressed that it would be fine even to ignore CHAMELEON's output completely.

**Figure 2:**
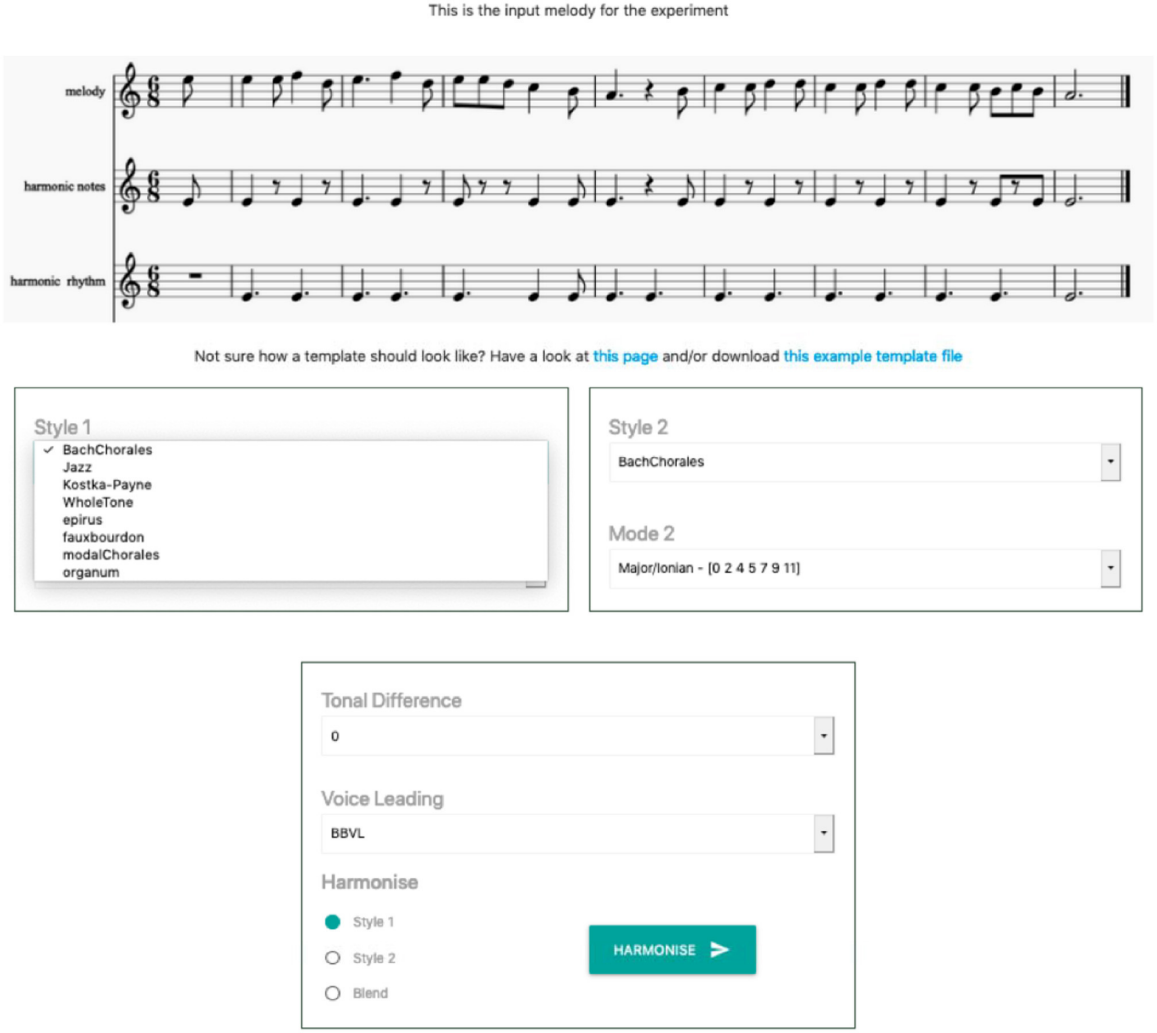
The online CHAMELEON user interface. The melody for harmonization together with the requested harmonic rhythm (i.e., when chord changes should occur) and important harmonic notes (i.e., notes that CHAMELEON will prioritize over others when selecting suitable chords for the underlying melody) were shown at the top. The options for harmonization were provided below. The user could select from the available harmonic styles using a drop-down menu and use the radio button at the bottom to opt for either a single-idiom harmonization or a blend between two different idioms. Some harmonic styles include more than one harmonic mode that the user could chose from. In addition, there was the option to blend two selected styles in different tonalities using the tonal difference drop-down menu. Finally, the interface allowed users to select type of voice leading. If no voice leading was necessary the system output chords in root position. The other option performed a rudimentary voice leading in the bass voice and in the intermediate voices.

Figure 2 shows the online CHAMELEON user interface that was provided to the participants. This interface allowed users to choose from the following eight harmonic idioms (styles) available in all modes for each idiom:

- Selection of 35 Bach chorales from the Breitkopf edition. This set represents baroque homophonic harmonic style (seventeenth-eighteenth century).
- Selection of jazz standards from the Real Book (melodies with chord symbols), mainstream jazz harmony.
- The dataset of the Kostka-Payne corpus (Kostka and Payne, 2013), produced by David Temperley (chord-list file) and Bryan Pardo (MIDI files with chords' quality). This set represents classical and romantic harmonic style (eighteenth-nineteenth century).
- Selected short excerpts of twentieth century whole-tone harmonization concepts from the textbooks of Stefan Kostka, Kent Williams, Walter Piston, and various other sources.
- Selection of 3-voice and 4-voice polyphonic songs from Epirus (transcriptions by K. Lolis), minor pentatonic harmony.
- Fauxbourdon excerpts or short pieces (thirteenth-fourteenth centuries, Dufay, Binchois, et al.).
- Selected modal homophonic chorales by Osiander, Praetorius, Scheidt, Hassler, Vulpius, Lasso, Walter, et al. Further categorization by mode is possible.
- Organum excerpts or short pieces (eleventh-twelfth centuries).

The participants had the additional option to blend two selected styles in different tonalities, as, for instance, blend C minor in Bach Chorale style with E major in the Jazz style (it is even possible to blend two different tonalities of the same style, e.g., blend C major with E♭ major in the Bach Chorale style). Two voice layout options were also offered: (a) root position implementation of chords (that made reading the output easier); and (b) a statistical learning-based method that learned and first applied bass voice leading (in case of blending the statistical models of the blended idioms were averaged) and then intermediate voices were placed in closed position under the melody. The voice leading method is based on Cambouropoulos (2015) and it employs a Hidden Markov Model for determining the bass voice leading given the current melodic motion, the previous position of the bass and an expected distance between bass and melody voices. Pressing the “harmonize” button revealed a basic notation-like representation of the requested harmonization and gave the options to play it back and download it in a MusicXML format.

In both experimental phases, participants filled in a 7-point Likert scale post-task questionnaire for user-experience evaluation whose questions represented the following metrics as defined by Kantosalo and Riihiaho (2019): Enjoyment, Expressivity, Outcome satisfaction, Ease of use, Collaboration, Ownership, Exploration, Immersion, and Productivity. Table 1 presents the questions corresponding to each metric both for the simple harmonization and for the computationally assisted task. Notice that some metrics did not apply to the simple harmonization task and were therefore not used. In addition, four free-answer questions concluded the questionnaire of the computationally-assisted harmonization (second phase). Apart from filling in the post-task questionnaires, participants submitted their melodic harmonization for each phase. For the computationally-assisted harmonization task they were given the option to submit up to four example harmonizations produced by CHAMELEON which had attracted their interest or even potentially influenced their own harmonization.

**Table 1:** Metrics (as general concepts) and corresponding questions for the post-task questionnaires.

**Metric**	**Quest. no**.	**7 point likert statement**	
		**Simple harmonization**	**Computationally supported harmonization**
Enjoyment	Q1	–	I would be interested to use CHAMELEON in the future
Expressiveness	Q2	The harmonization of a given melody gave me the opportunity to be creative	The use of CHAMELEON gave me the opportunity to be creative
	Q3	I was able to express my ideas	I was able to express my ideas
Outcome satisfaction	Q4	I am satisfied with my harmonization	I am satisfied with my harmonization
Ease of use	Q5	The process of harmonization was easy	The process of harmonization was easy
	Q6	–	The use of CHAMELEON was easily comprehensible
Collaboration	Q7	–	CHAMELEON provided me with some good ideas
	Q8	–	CHAMELEON provided me with some good solutions that I could not have reached myself
Ownership	Q9	I feel that the harmonization belongs to me 100%	I feel that the harmonization belongs to me 100%
Exploration	Q10	–	The contact with CHAMELEON offered me a different harmonization perspective
Immersion	Q11	I was able to maintain concentration on my task	The use of CHAMELEON helped me maintain concentration on my task
Productivity	Q12	I was productive	CHAMELEON affected my productivity positively
Free-answer questions		–	What did you like the most about CHAMELEON?
		–	What did you like the least about CHAMELEON?
		–	If you had the opportunity which of the system's capabilities would you redesign?
		–	What are your thoughts regarding computationally assisted music composition after this experience?

### 2.2. Control Experiment

The presentation order of the tasks in the main experiment remained fixed since the simple harmonization experience of melody A was viewed as a reference for the user-experience evaluation of the computationally supported harmonization of melody B. Therefore, a possible order effect needed to be taken into account for the analysis of extracted harmonic features (see section 2.3). To this end, a complementary control experiment was conducted whereby the two harmonizations were performed in the same fixed order (melody A first, followed by melody B) but without computational support for the second task. Twenty two students from the School of Music Studies of the Aristotle University of Thessaloniki (*N* = 22, mean age = 20.3, std age = 5, 12 female) took part in the control experiment. The directions were identical to the main experiment but participants were not asked to fill in questionnaires for user-experience evaluation. Only the two produced harmonizations were acquired. This design examines whether potential differences in the characteristics of the harmonizations acquired through the two experimental conditions could be attributed to the treatment (i.e., computational support) or resulted from other factors.

These experiments were certified for ethical compliance by the research ethics board of the Aristotle University of Thessaloniki (Ref. number 66117/2020).

### 2.3. Calculation of Harmonic Features

The subjective user experience evaluation was complemented by a more objective assessment of harmonization characteristics based on computational extraction of harmonic features. Given that participants were free to use any type of harmonic palette, thus potentially avoiding tonal harmonic devices, harmonic content had to be captured using idiom-independent features of harmonic plurality. To this end, three different features based on the General Chord Type (GCT) (De Haas et al., 2008), the isolated type component of GCTs (without root information) and the Pitch Class Profiles (PCPs) were extracted from each harmonization. The plurality of harmonic content within a harmonization was quantified through the absolute number of GCT chords (unique root-type components) and chord types (isolated type component of the GCT). The PCP is the 12-dimensional vector that describes the percentages of pitch classes in the entire harmonization (harmonic part without the melody). To obtain the complexity of a harmonization based on its PCP, the Shannon Information Entropy (SIE) of this distribution was computed, allowing the representation of any harmonization complexity through a single numerical value (Kaliakatsos-Papakostas et al., 2010); the following formula is employed: $\text{SIE}=-\underset{p}{\sum }\text{PCP}\left(p\right)\;\log\left[\text{PCP}\left(p\right)\right]$, where PCP(p) is the distribution value for pitch class *p*. Greater SIE values indicate PCP distributions that are more uniform, which, in turn, indicates a richer variety of pitch content in the harmonization. Therefore, each harmonization was described by these three values, for each of which higher values indicated more complex harmonizations.

Apart from capturing complexity we were also interested in quantifying the diversity of the harmonizations obtained for each experimental condition together with their deviance from typicality. A prerequisite for this was the ability to calculate distances between harmonizations. The feature values were used to estimate such distances between harmonizations (see numbered list below) which were subsequently compared between conditions. These distances were also exploited to construct geometric representations of the harmonizations through Multidimensional Scaling Analysis (MDS). Specifically, distance metrics between harmonization 1 (*h1*) and harmonization 2 (*h2*) were devised as follows:

1. Common GCTs: the number of non-common GCTs employed in *h1* and *h2* over the number of total unique GCTs in *h1* and *h2*. In other words, this metric shows how many non-common chord labels are used in the two harmonizations; the larger the number, the higher the dissimilarity.
2. Common chord types: as above but restricted to the type component of the GCTs (regardless of root). This metric incorporates the types or “qualities” of the chord labels—e.g., in jazz guitar-style chord notations, how many non-common X7 or Xm7 are included in the harmonizations under comparison.
3. PCP distance: 1 minus the correlation of the (12-dimensional) PCP vectors (distribution of pitch classes throughout the entire harmonization) extracted from *h1* and *h2*. Since the compared melodies are transposed to the same key, PCP vectors are unbiased in terms of tonality. This metric indicates the similarity of the overall harmonic content between two harmonizations. PCP information has proven efficient for categorizing music according to style (Kaliakatsos-Papakostas et al., 2010).

## 3. Results

### 3.1. Fixed Creativity Metrics

#### 3.1.1. Assessment of the Overall Influence on Creativity for Composers and Students

**Figure 3:**
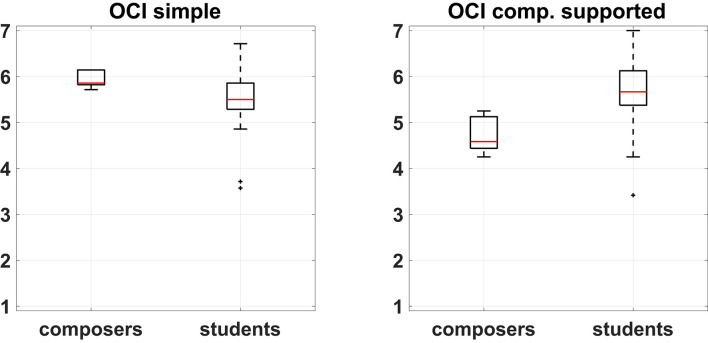
Boxplots of the Overall Creativity Index (OCI) in the two experimental conditions for both participant groups. Mean OCI simple for composers = 5.8, Mean OCI simple for students = 5.57, mean OCI comp. supported for composers = 4.7, mean OCI comp. supported for students = 5.6.

The data of the post-task questionnaires were initially used to provide an indication of the overall creativity support offered by CHAMELEON as assessed by the two participant groups (students and composers). This was quantified by a metric that we refer to as the Overall Creativity Index (OCI) which was estimated as the average score of all the post-task questions for each participant. Figure 3 shows the OCI boxplots between composers and students for the two experimental conditions. Since the OCI is derived from averaging ordinal data (i.e., ratings on Likert scales), the comparison of the OCI between the two groups was made through the non-parametric Wilcoxon rank-sum test. Results showed that means do not differ significantly (*z* = −2, *p* = 0.051) between the two groups in the simple harmonization, albeit the *p*-value is marginally above the 0.05 threshold. On the other hand, there is a significant difference (*z* = 2.31, *p* = 0.042, effect size = 0.33, median difference = 1.08) in the computationally supported case. This constitutes a reversal of the picture between the two conditions. Indeed, in the simple harmonization composers featured a higher OCI (even though marginally not significant enough), but in the computationally supported task their OCI declined substantially and was significantly lower than the corresponding OCI of students. It should be noted that the two conditions are not directly comparable at the general level due to the different number of questions involved in each post-task evaluation. These results already indicate an overall trend in the assessment of CHAMELEON between groups of participants and the following subsections will examine which specific questions are responsible for this general picture.

#### 3.1.2. Comparison Between Experimental Conditions for Each Group

**Figure 4:**
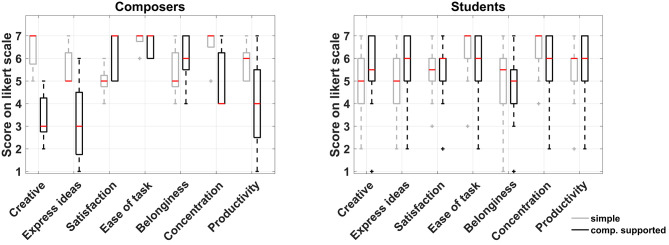
Boxplots of the seven common questions between the two experimental conditions for composers and students.

Figure 4 shows the box plot for the responses to the seven common questions between the two experimental conditions for the two participant groups separately. Again, a non-parametric approach was adopted for the comparison of the medians due to the ordinal nature of the data. The small sample size of the composers' group prevented the identification of significant differences in any of the questions, although some of the differences observed in the medians between the two conditions are quite large but still not necessarily significant. On the other hand, the students' group featured significant differences in three instances as shown in Table 2. Based on the results of the Wilcoxon signed-rank test, they felt it was easier to express their ideas when supported by CHAMELEON but at the same time this task was regarded as more difficult and it was harder for them to maintain concentration while using CHAMELEON. At this point, it has to be noted that effects in this study will not be corrected for multiple comparisons. If the level of significance was reduced to *p*/7 in this case (taking into account the 7 paired comparisons) according to a Bonferroni correction, then no effects would be identified. However, such an approach would increase the probability of falling into a type II error and rejecting an existing effect. According to the guidelines by Armstrong (2014) regarding the appropriate use of the Bonferroni correction, a study that includes only a small number of planned comparisons should not correct for multiple statistical testing. Since our study satisfies this condition, all current results will be reported at the significance level of *p* < 0.05.

**Table 2:** Statistically significant differences that resulted from the Wilcoxon signed-rank test between the responses of the two experimental conditions for the students' group.

**Question**	***z***	***p*-value**	**Effect size**	**Median difference**
Express ideas	−2.2	0.028	−0.35	−1
Ease of task	2.3	0.023	0.37	1
Concentration	2.6	<0.01	0.41	1

*No statistically significant differences were identified for the composers' group*.

*Effect size: ($r=\frac{z}{\sqrt{N}}$)*.

#### 3.1.3. Comparison Between Groups on Each Task

**Figure 5:**
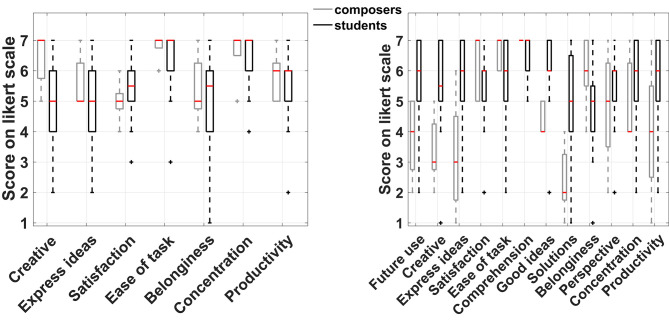
Boxplots of the responses on each task between groups of participants.

The responses did not pass the Shapiro-Wilk test for normality and as a result a non-parametric approach for median comparison was followed. Only one statistically significant difference was identified for the simple harmonization task and this was on the statement: “The harmonization of a given melody gave me the opportunity to be creative” with which composers agree more (Wilcoxon rank-sum test: *z* = −2.2, *p* = 0.03, effect size = 0.44, median difference = −2). On the contrary, the computationally supported condition featured a number of statistically significant differences. Table 3 shows the statistically significant differences that resulted from the Wilcoxon rank-sum test between the two groups for the computationally supported task and Figure 5 presents the respective boxplots. These differences indicate that the use of CHAMELEON resulted in higher enjoyment, higher capability of expressiveness and stronger collaboration with the system for the students' group than for the composers' group.

**Table 3:** Results of the Wilcoxon rank-sum test between the two groups for the computationally supported task.

**Question**	***z***	***p*-value**	**Effect size**	**Median difference**
Future use	2.6	<0.01	0.37	2
Creative	2.6	<0.01	0.37	2.5
Express ideas	2.4	0.01	0.34	3
Good ideas	2.6	<0.01	0.37	2
Solutions	2.9	<0.01	0.41	3

*Effect size: ($r=\frac{z}{\sqrt{N}}$)*.

### 3.2. Free Text Evaluation

Participants also provided free-text answers to four post-task questions regarding CHAMELEON's use. Table 4 summarizes opinions expressed by the two groups. The free-text responses showed that harmonic blending was the most appreciated system capability whereas the problematic score visualization with mistakenly spelled enharmonic notes and many ledger lines was identified as the major weakness. In addition, some participants would like to have been offered a greater variety of harmonic idioms and more convincing harmonizations in some styles. With regard to their thoughts on computationally supported music composition, the majority of the students mentioned that a computational assistant can potentially offer new ideas, promote productivity and increase creativity. However, some of them were not very keen on embracing its use stating that music creation should be exclusively a human endeavor. Additionally, a couple of composers expressed the opinion that computational assistance is more suited for amateur musicians.

**Table 4:** Summary of the free text answers provided by the participants to the four post-task questions regarding the computationally supported task.

**Group**	**Questions**			
	**What did you like the most about CHAMELEON?**	**What did you like the least about CHAMELEON?**	**What feature of CHAMELEON would you redesign if you had the opportunity?**	**What are your thoughts regarding computational support in music composition after this experience?**
Composers	The capability of harmonic blending (3), the playback possibility	The lack of voice leading (2), the visualization of the produced score (2)	Some of the harmonic idioms which are not very convincing style-wise (2), more harmonic styles and scales (2)	Mostly for amateurs (2), can save time and provide ideas, computer creates the possibilities among which a composer can select
Students	The capability of harmonic blending (8), diverse solutions (5), ease of use-speed-playback (4), gives you ideas (3), saves you time	The visualization of the produced score was hard to read (7), the limited harmonic idioms available (5), the resulting harmonizations (4)	Increase the available harmonic idioms (6), make scores easier to read (3), include harmonic (2) analysis	Can give rise to new ideas (8), can increase productivity (5), can promote creativity (3), music composition should be an exclusively human endeavor (4), useful up to a point (3)

*Numbers in parentheses indicate the number of participants that provided a similar answer*.

### 3.3. Comparison Between Harmonizations of the Main and the Control Experiment

The previous section dealt with evaluating user experience of the computationally supported harmonization. This section will compare the produced harmonizations between the main and the control experiment utilizing the harmonic features described in section 2.3.

#### 3.3.1. Comparison of Complexity

**Figure 6:**
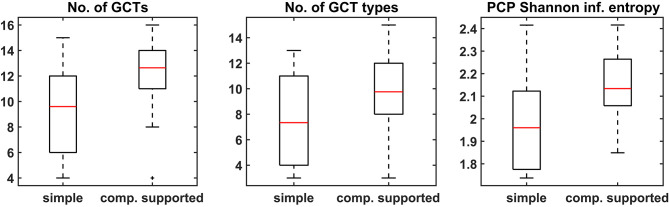
Boxplots of the three harmonic features for both the simple and the computationally supported task.

All three harmonic features (number of GCTs, number of GCT types, and PCP) that were calculated indicate an increase in the harmonic complexity of harmonizations when musicians are computationally assisted by CHAMELEON in comparison to the simple harmonization task. Figure 6 shows that the number of GCTs as well as the number of GCT types were higher in the second task. The SIE of the PCP was also higher, indicating a flatter distribution of pitch classes in the second task. The medians of all metrics for the main experiment (computational support) are significantly greater compared to the control group (at significance level *p* < 0.05), as shown in Table 5 that presents the results of the Wilcoxon sign-rank tests. At the same time, for the control experiment, only the number of GCTs is significantly increased in the second condition but with a comparatively smaller effect size (−0.32 to −0.52). To make between group comparisons, we calculated distance vectors by taking the difference of each feature between the two experimental conditions (e.g., number of GCTs in the harmonization of melody A - number of GCTs in the harmonization of melody B). These vectors were subjected to Wilcoxon rank-sum tests for independent samples which showed that the differences were significantly greater for the main experiment in comparison to the control experiment for all metrics of harmonic complexity.

**Table 5:** Within and between group comparisons of the harmonizations obtained from the main and the control experiment based on three different harmonic features.

		**Computational support (within group)**				**Control (within group)**				**Distance vectors (between groups)**			
	**Metric**	***z***	***p*-value**	**Effect size**	**Median diff. A-B**	***z***	***p*-value**	**Effect size**	**Median diff. A-B**	***z***	***p*-value**	**Effect size**	**Median diff. A-B**
Complexity	GCTs	−3.68	**<.001**	−0.52	−3	−2.12	**0.017**	−0.32	−1	−1.85	**0.032**	−0.27	−1
	GCT types	−2.94	**0.002**	−0.41	−2.4	−1.13	0.13	−0.17	−1	−1.86	**0.031**	−0.27	−2
	SIE of PCP	−3.83	<**0.001**	−0.54	−0.17	−1.36	0.086	−0.20	−0.06	−2.99	**0.003**	−0.44	−0.12
Diversity	GCTs	−6.85	<**0.001**	−0.28	−0.01	−2.85	**0.002**	−0.13	0	−2.76	**0.003**	−0.12	−0.0023
	GCT types	−2.92	**0.002**	−0.12	0	−2.16	**0.015**	−0.10	−0.02	−1.4	0.076	−0.061	−0.0208
	SIE of PCP	−4.46	<**0.001**	−0.18	−0.03	2.26	0.99	0.10	0.01	−5.77	<**0.001**	−0.25	−0.0408
Unexpectedness	GCTs	−2.64	**0.004**	−0.37	−0.03	−1.5	0.066	−0.23	−0.03	−5.86	<**0.001**	−0.85	−0.94
	GCT types	−1.83	**0.034**	−0.26	−0.05	−1.31	0.096	−0.20	−0.02	−5.86	<**0.001**	−0.85	−0.91
	SIE of PCP	−1.67	**0.047**	−0.24	−0.01	−0.027	0.393	−0.04	0.01	−5.85	<**0.001**	−0.85	−0.61

*Wilcoxon signed-rank tests were employed to compare melodic harmonizations A and B for harmonic complexity, diversity, and unexpectedness (for definitions of these concepts please see the text) within each experimental group (main and control). Wilcoxon rank-sum tests were employed to directly compare between the independent experiments utilizing a vector of distances between harmonizations of melodies A and B (based on the harmonic features). The p-values in bold represent statistically significant effects at the level of 0.05*.

*Effect size: ($r=\frac{z}{\sqrt{N}}$)*.

#### 3.3.2. Comparison of Diversity

The higher values in the harmonization metrics reported above are an indication of increased complexity of the harmonizations produced with the assistance of CHAMELEON. We further calculated the distances between harmonizations based on these metrics to assess a potential difference in the divergence of the outcomes between the two experimental conditions. The distances between harmonizations were calculated as described in section 2.3.

**Figure 7:**
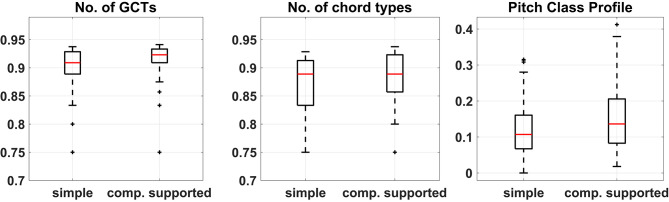
Boxplots of the differences between harmonizations calculated based on the three features for the two conditions of the experiment. Median difference in No. of GCTs = −0.014, No. of chord types = 0, and Pitch Class Profiles = −0.029.

The vectors of distances for all metrics did not pass the Shapiro-Wilk test for normality and therefore their medians were compared using the non-parametric Wilcoxon rank-sum test. All comparisons were significantly lower (at the *p* = 0.05 level) for the simple harmonization task compared to the computationally supported one, thus indicating that the outcomes of the second condition were less homogeneous. Figure 7 shows the boxplots of the distances among harmonizations based on the three features. Again, Table 5 shows that the computationally supported condition features significantly greater medians on all metrics (at the *p* = 0.05 level) according to the Wilcoxon sign-rank tests. In this case, however, the control experiment also features higher medians in two out of the three metrics, although with smaller effect size for the number of GCTs. The direct comparison between groups shows that larger diversity of produced harmonizations may be supported only based on the number of GCTs and the SIE of the PCPs, and not on the number of GCT types.

#### 3.3.3. Comparison of Unexpectedness

**Figure 8:**
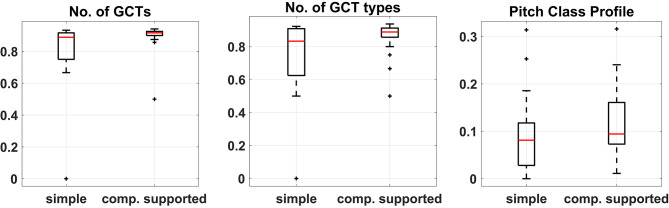
Boxplots of the differences between harmonizations and the typical solution calculated based on the three features for the two conditions of the experiment. Median difference in No. of GCTs = −0.028, No. of chord types = −0.056, and Pitch Class Profiles = −0.013.

To measure the degree of departure from the most expected harmonic solutions we asked a professor of music theory to create the most typical harmonizations as implied by each of the two melodies. These harmonizations are presented in the Supplementary Material. Subsequently, the distances between each of these two reference harmonizations and the corresponding participants' solutions were calculated based upon each of the three harmonic features and the distributions of the two vector distances were compared. A Wilcoxon rank-sum test was performed for each of the three feature-based distance pairs because all of them failed to pass the Shapiro-Wilk test for normality. These tests showed that in all three cases, the median of the distances between the harmonizations created by the participants and the corresponding most expected (implied) harmonization was significantly higher in the case of computational support than in the unsupported case. This indicates that, on average, participants were moving further away from the most typical harmonic solution when interacting with CHAMELEON in comparison to when harmonizing on their own. Figure 8 shows the boxplots of the distances between harmonizations for each condition and the corresponding typical solution. Such differences were not observed in the control experiment in any of the metrics. In addition, the direct between-groups comparison showed that participants in the main experiment were drawn further away from the most typical harmonizations in comparison to the control group.

**Figure 9:**
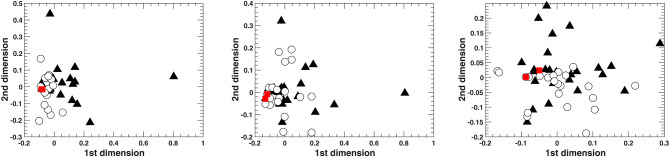
Two-dimensional spatial configurations from Multidimensional Scaling on the distances based on **(left)** the number of GCTs, **(middle)** the number of GCT types, **(right)** the pitch class profiles. The red squares represent the implied harmonizations for the two melodies, the white circles represent the 25 simple harmonizations and the black triangles represent the 25 harmonizations that resulted from the computationally supported task.

The above is also evident from Figure 9 that presents the spatial configurations of the relationships between all the harmonizations based on the three different harmonic features (number of GCTs, number of GCT types, and PCP). The two-dimensional spatial configurations were calculated through a non-metric Multidimensional Scaling (MDS) analysis. It has to be noted that, for the number of GCTs and GCT types, the MDS model was very good for two dimensions (Stress1 = 0.08 and 0.13, respectively) whereas the model for PCP was poor (Stress1 = 0.30). Stress1 is a measure of misfit where a lower value indicates a better fit between actual and estimated distances (with a minimum of zero). One has to bear in mind that the relationships between harmonizations in these figures are represented by a mere two-dimensional model approximating the actual distances calculated by the harmonic features. However, it is clear from these representations that the two implied harmonizations are very similar to each other and that the simple harmonizations (white circles) tend to cluster closer to them than the computationally supported ones (black triangles). This is less prominent in the PCP-calculated distances, where the model is the least adequate for two dimensions.

## 4. Discussion

The experiment presented in this paper aimed to study the potential influence of a creativity support tool in music composition. The analysis of both behavioral metrics of creativity and computationally extracted descriptive features of the obtained harmonizations revealed some interesting findings regarding the use of CHAMELEON by the participants in this study. The general picture is that students seemed to appreciate the use of this tool more in comparison to professional composers based on the behavioral responses (see Figure 3 and Table 3). More specifically, students appreciated CHAMELEON's assistance in expressing their ideas better despite finding it more cognitively demanding (see Table 2) compared to the simple harmonization. This did not prevent them from being significantly more favorable than composers regarding the future use of the tool (see Table 3).

The free-text responses indicated that the harmonic blending capability of CHAMELEON was appreciated by the participants but also highlighted that the visualization of the score was considered very primitive and thus requires re-designing. Interestingly, the views of participants on computationally supported creativity based on this particular experience varied. It was quite evident that the sophistication of the system did not match the sophistication possessed by professional composers on a melodic harmonization task and as a result they were not really impressed by its use. On the contrary, a large number of students seemed a lot more enthusiastic regarding the prospect of computational support in music creation based on their experience. Of course, free responses varied even within the student group probably reflecting different levels of expertise in melodic harmonization and even different levels of biases regarding the use of computational tools as creative assistants in music making.

The fixed-scale and free questionnaires measured aspects of how the system was perceived by its users and helped us identify features that were deemed stronger or weaker. Additionally, the computational analysis of the harmonizations complemented the picture of the behavioral data since this analysis did not involve the subjective judgement of the users. Harmonizations before and after computational support were quantitatively compared in terms of three concepts that usually appear in creativity evaluation literature: *complexity, diversity*, and *unexpectedness*. These comparisons revealed statistically significant differences between the two conditions of the main experiment. Computationally assisted harmonizations were more complex and unexpected overall in addition to forming a more diverse group.

To ensure that the effects identified from the main experiment should be attributed to the influence of CHAMELEON and not to the mere repetition of a similar task we examined the above findings in comparison with the corresponding ones from the control experiment. In all cases (with the exception of the metric number of GCT types for diversity) all kinds of comparisons between the control and the main experiment showed that the differences in harmonic complexity, diversity, and unexpectedness were greater for the main experiment compared to the control experiment. In other words, the increase of complexity, diversity, and unexpectedness observed in the computationally supported condition cannot be fully accounted for by the mere repetition of the task and should indeed be attributed—in some cases fully, in some partially—to the interaction with CHAMELEON.

These results show that a system for computational support had a clear and quantifiable influence on the harmonizations created by human users. The question that arises is whether this observation could suggest an increase in the creativity of the users. The mere increase in complexity that was observed in the computationally supported artifacts is not sufficient to justify such a claim as the relationship between perceived value and complexity is not linear but is best modeled through the bell-shaped Wundt curve (Heyduk, 1975). This means that when it comes to complexity there exists an optimal level that leads to the highest appreciation. However, we asked our participants to submit their harmonizations based solely on satisfaction of their personal preference (even at the cost of ignoring completely all the suggestions provided by CHAMELEON). This way it was ensured that the computationally supported harmonization would possess at least equal aesthetic value compared to the unsupported one as judged by their creator. Indeed, it is reasonable to assume that the computationally supported harmonization—which was always created last—could not have been deemed inferior to the simple one, given that participants were absolutely free to stick to their initial harmonic solution. That is, if participants chose to substantially alter their first approach this should indicate that they favored the altered version equally (if not more). According to our working definition of creativity presented in the introduction, the above rationale essentially transforms the evaluation of creativity to an assessment of novelty with respect to the produced artifacts. The fact that the computationally supported harmonizations featured higher complexity, higher originality and constituted a more diverse group compared to the unsupported ones implies that at least some users must have exhibited some form of P-creativity (i.e., they produced something that was novel for themselves but not necessarily novel to the world). This is also supported by the behavioral ratings on the statement: CHAMELEON provided me with some solutions that “I could not have reached myself” that received a median value of five out of seven in the students group.

At this point, it is important to note that based on this experimental design it is hard to argue that computational support actually increased the creativity of the process (i.e., the manner by which users reached solutions). Furthermore, the press/environment and producer components of the four Ps framework (Jordanous, 2016) were deemed constant in our case and were thus not considered at all. However, the presented evidence indicates that computational support can potentially affect the properties of the products by transfusing characteristics from which humans tend to extrapolate higher (perceived) creativity. All things considered, this study revealed that computational support inspired some users to be more adventurous and explore new harmonic spaces away from the most obvious harmonic solutions. At the same time, it should be acknowledged that this evidence stems from a case study with certain characteristics, the most notable of which is the fact that the requested task was deliberately chosen to be very simple. The selected melodies for harmonization featured strongly implied, simple harmonies in both experimental conditions and the implicit research question was: “can an otherwise simple harmonization task be affected by the suggestions of a melodic harmonization assistant?” It may be assumed that had the task been more complex and with higher degrees of freedom to begin with, (i.e., loosely implied harmony and high melodic chromaticism) the influence of computational support would have been less pronounced as users would tend to produce more complex and unexpected outcomes even without computational support. Different scenarios should also be examined in future work to obtain a more comprehensive perspective regarding the influence of CHAMELEON in melodic harmonization.

The experiments presented in this paper attempted to simulate natural conditions of music making as much as possible; however, it could not avoid certain restrictions in order to maintain a controlled experimental procedure, such as strict guidelines regarding harmonic rhythm and texture. As an additional qualitative exploration of the use of CHAMELEON, a further project was encouraged in the domain of free, unrestricted compositional practice. Six of our participants with varying levels of compositional experience volunteered to create musical vignettes for a string quartet inspired by our experiment. They used the melody of the “Lullaby from Southern Italy” (melody B) as primary material and drew upon harmonic information produced by CHAMELEON. These original works were presented in a concert in which the composers explained to the audience how they employed the creativity support system in their work. The creative exploitation of ideas and concepts suggested by CHAMELEON was evident in each of the short compositions and two indicative examples are presented below.

**Figure 10:**
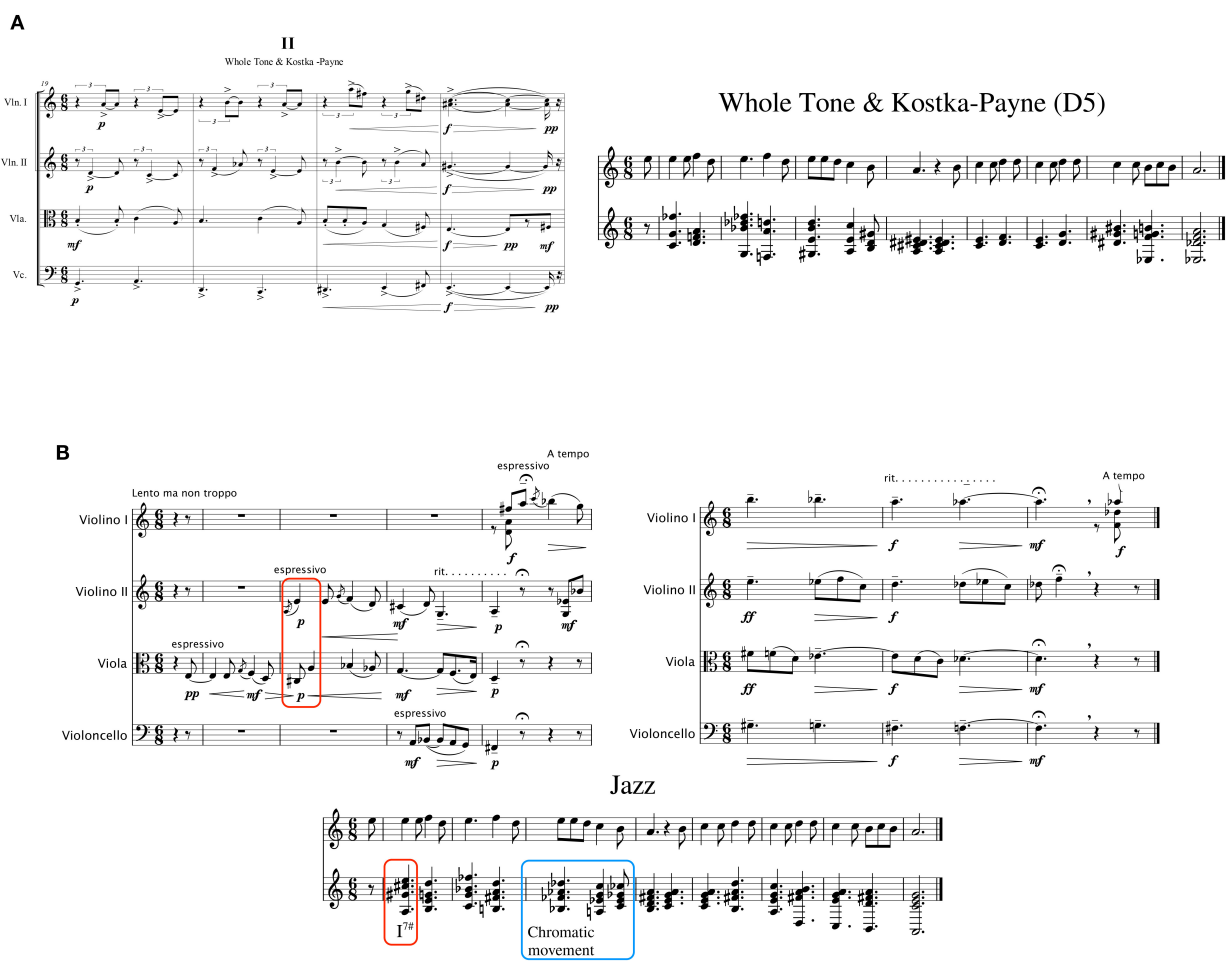
**(A)** Left: the beginning of the second variation of the original piece; right: the Whole Tone and Kostka-Pane blend with five semitones tonal difference by CHAMELEON. The harmonic sequence is transposed one perfect fifth up from Am to Em and although there are differences in the spelling of enharmonic notes, in voice leading and some added notes, the harmonic backbone remains the same as the one suggested by CHAMELEON. **(B)** Top left: the beginning of the original piece; top right: measures 23–25; bottom: the Jazz harmonization by CHAMELEON. It can be seen that the tonic major opening chord (I) also appears in the Jazz harmonization as a I^7♯^. The 3rd measure of the Jazz harmonization contains a downward chromatic movement in the three top voices that was adopted by the composer in measures 23–25 of his composition.

One of the composers created five short variations each named after one CHAMELEON harmonic blend, namely: I. Bach chorales & Organum; II. Whole Tone & Kostka-Pane; III. Epirus & Jazz; IV. Kostka-Pane & Jazz; V. Modal chorales & Kostka-Pane; thus indicating the harmonic influence from each one of these blends. The first four measures of the second Whole Tone & Kostka-Pane variation are presented in Figure 10A next to the harmonization by CHAMELEON from which it originated. The composer informed us that she transposed the harmonic sequence given by CHAMELEON one fifth up (from Am to Em), but apart from adding some extra notes and voice leading, she remained faithful to the backbone harmonic sequence as output by CHAMELEON. Another composer (Figure 10B) borrowed an idea from the Jazz CHAMELEON harmonization, that opens the piece with a tonic major (I) chord rather than a tonic minor (i) chord that would be the norm in a minor mode. He also informed us that he used the tonic major in various instances throughout his piece. One other example of influence in this piece was the adoption of a downward chromatic movement in the three upper voices similar to the 3rd measure of the Jazz harmonization by CHAMELEON.

These examples are only indicative of the many different harmonic possibilities and ideas suggested by CHAMELEON and adopted by the composers in their final works and they demonstrate the potential of human-computer collaborations in music creation. It is important to note that these influences were pointed out by the composers themselves and were not identified through our own analysis. On the contrary, and back to our main experiment, we recently presented a comparative analysis between the computationally supported harmonizations and the favored CHAMELEON examples of each participant that identified a number of different strategies for the creative exploitation of CHAMELEON (Zacharakis et al., 2020) in a melodic harmonization task. Indeed, many participants seemed to have adopted elements as they appeared in their preferred CHAMELEON examples ranging from single chords (most usual) or longer chord sequences to more abstract concepts (less usual).

## 5. Conclusions

Through a combination of user experience assessment and computational characterization of the produced harmonizations it has been shown that the use of CHAMELEON resulted in more explorative approaches on a melodic harmonization task, but was appreciated more by novices than experienced composers. At the same time, novel musical compositions inspired by this experiment featured clear influences by CHAMELEON as reported by their own creators. The feedback received from this experiment will be utilized in future versions of the harmonization assistant to improve user experience. Although participants reported that the use of CHAMELEON was easily understandable, they seem to refer primarily to the ability to choose and combine its parameters rather than the understanding of its background processes. The system in its current form is most likely interpreted as a *black box* with unspecified internal processes. It is possible that adding layers of explainability could increase the sense of collaboration between the users and the machine which may in turn increase the sense of overall enjoyment. In addition, transparency regarding how and why the system reaches particular solutions might mitigate beliefs—such as the ones expressed in this study—that consider artificial intelligence as an inappropriate medium for music composition. Future work will investigate the influence of such added features to the experience of using a creative music assistant.

## Data Availability Statement

The raw data supporting the conclusions of this article will be made available by the authors, without undue reservation.

## Ethics Statement

The studies involving human participants were reviewed and approved by the Research Ethics Committee of the Aristotle University of Thessaloniki. The patients/participants provided their written informed consent to participate in this study. Written informed consent was obtained from the individual(s) for the publication of any potentially identifiable images or data included in this article.

## Author Contributions

AZ, MK-P, SK, and EC participated in the conception and design of the study. AZ and SK conducted the experiments. MK-P created the online version of CHAMELEON and extracted the harmonic features from the obtained melodic harmonizations. AZ performed the statistical analysis. All authors contributed to the writing of the paper, manuscript revision, and approval of the submitted version.

## Conflict of Interest

The authors declare that the research was conducted in the absence of any commercial or financial relationships that could be construed as a potential conflict of interest.

## Footnotes

^1^https://github.com/maximoskp/chameleon

^2^CHAMELEON, as a basic melodic harmonization assistant, receives a melody as an input and suggests chord symbols for harmonizing this melody. It also generates actual chords that implement those chord symbols, but with rudimentary voice leading without expressional characteristics, i.e., vertical chords are placed whenever chords change. Therefore, CHAMELEON does not create actual arrangements as, for example, “Band In A Box,” but it automatically fills in the chords to be played by an arrangement (e.g., preparing a chord chart for a “Band In A Box” song).

## Supplementary Material

Supplementary Figure 1 The most typical harmonizations of the two melodies used in the experiment as indicated by a professor of music theory.
